# Popular Education in Dietetic Economics

**Published:** 1919-07

**Authors:** A. L. Benedict

**Affiliations:** Buffalo, N. Y.


### POPULAR EDUCATION IN DIETETIC ECONOMICS

BY A. L. BENEDICT, A.M., M.D., BUFFALO, N. Y.

In view of the various propagandas of popular nature, it may be allowable to suggest that one more should be undertaken, especially since no apology has been offered for systematic instruction of the laity in regard to the early diagnosis and general principles of treatment of cancer, about which the medical profession still is in almost absolute ignorance. It is doubtful whether a special philanthropic organization should be formed for this purpose, only further to tax the benevolent and to maintain working staffs and to increase the revenue of paper manufacturers, printers and the post office. Still, if the medical profession and existing organizations for educational or philanthropic purposes take up the subject seriously and deal with it intermittently, as occasion may offer, much can be accomplished, perhaps all that need be desired; for, the people are eager for instruction, and the existing periodicals, from newspapers to professional journals, are ready to publish educational articles.

It is not intended in this article to go beyond some general principles and a tentative discussion of a general plan of popular education that would have to be elaborated by criticism and expert knowledge along various practical lines before it could be made workable.

Let us begin with the optimistic fact that this country of ours produces considerably more than the food required by its present population, and that production can be increased by methods at present practicable, so as to support at least five times its present population, merely by proper adjustment and putting known means into effect. An increase of population, especially if distributed so as to bring the density of parts at present scantily populated up toward the average, will increase the cost of certain foods, especially of meats, while, if the population is increased by the growth of cities, transportation and distribution expenses will increase the cost of most commodities, especially those requiring prompt delivery and care in handling, as, for example, milk, fresh fruits and vegetables.

### LAW OF SUPPLY AND DEMAND NOT CONTROLLING

Not much can be expected from the law of supply and demand as ordinarily understood. On the contrary, it usually works in an opposite way. For example, an oversupply tends to reduce the price, the consumer benefiting temporarily by getting bargains; production thereupon is discouraged and the price rebounds, often beyond its normal level. On the other hand, excess of demand raises the price, stimulates production, renders economy feasible in production and distribution, lessens losses from lack of sales, and, yet, through these same causes ultimately tends to reduce price permanently.

But, subject to possible economies in production, transportation, and so on, and the general business principle, which even telephone companies began to learn before the war—that it is better to sell a good deal of a commodity at a reasonable price than only a little at a fancy price—price will ultimately be controlled, not, by supply, but by demand—the demand of the various forms of capital and labor involved in production and distribution —for, as great return as can be got from any other form of industry, by the particular person or firm concerned or by those considered as analogous with regard to earning power. Perhaps it would be more accurate to say that each individual concerned wants a little more than he is worth or than his analog is getting.

In plain words, food will be priced upon the same general basis as plumbing, carpentry, gasoline distribution, brokerage or any other industry, with due reference to the earning capacity of those concerned. As food is an urgent necessity and as the ultimate producer is rather better circumstanced than are most workers as to a home, a supply of longlasting necessities, and the ability to feed himself and his family, in other words, is assured to a large degree against actual want in anything that may be compared to a strike, the consumer is really more at the mercy of the factor of demand with respect to food than to any other commodity.

Of course, the food consumer would like to see his own wages or salary or profits go up and those of the farmer and his hired man and of others engaged in food production and distribution keep at the former standards. Just as much wheat or potatoes will grow on an acre as formerly, rather more; it requires no more acreage to produce a gallon of milk than formerly, possibly less; chickens can pick up their food and the farmer's children can pick up the eggs, they can be put into a basket of oats and sold at a cent apiece just as formerly. The coffee, tea, sugar, tinware, calico, and so on, which the farmer had to buy, .cost no more than formerly, even at war prices, and their normal price is considerably less. There really is no reason why food should go up in price, except for the wishes and demands of the man that produces it. For that matter, any one that is solely a consumer of food can go back to the farm and have the major part of his sustenance at a cost too small to be counted at all, if he chooses. Why doesn't he?

### THE MIDDLEMAN IS NEEDED

The elimination of the middleman has been suggested as a panacea. The technical objections to this can be better treated by more expert economists; still, some practical illustrations of what would result may here be suggested. The next time, when some cog or chain or other intermediate part of your automobile gives trouble, take it out. After you have had it fixed and put back again, drive out into the country and buy a bushel of potatoes or a dozen of eggs or a pound of butter at a farm house

—if there is one accessible and the owner wants to be bothered with your small purchase. Then go to the market and see how much you have saved or lost. Or, in a more general way, try any of the producer-to-family schemes, and, with due regard to quality, calculate how much of the middleman's profit you get. One minor, although extremely important practical point is, that the producer—farmer or otherwise—reads the papers and knows just what the market price chances to be. And, he insists on getting it, too.

There are, undoubtedly, extortionate and dishonest practices by middlemen. So there are on the part of retailers, transporters, producers and consumers. All those should be dealt with rigidly and impartially, as all tend to increase the cost of living.

### FAIRNESS TO ALL REQUIRED

We are just beginning to recognize the fact that, while "*caveat emptor*" is a high-sounding and ancient injunction, it does not represent a principle either of ethics, law or economics. Nevertheless, we should not go to the opposite extreme and disregard the rights of the seller. The rights of everybody should be equally and equitably enforced. They must either be enforced by existing machinery of government, extended as necessary, or a government within a government, designed to deal informally with economic questions, must be established. It is questionable whether the reluctance to extend the domain of government so as to insure to each individual adequate return for his labor and to insure his fellows against his getting an excessive return, should be carried to the extent either of maintaining existing economic evils or of establishing a secondary union of people, to secure their economic rights by some form of economic coercion.

It should be recognized, however, that experiments with consumers' leagues for the most part have failed and that similar combinations of producers, to eliminate the middleman and retailer have, if successful, not tended to the welfare of the consumer—oranges, for example.

An even broader view of the economic problem involved is necessary. The great bulk of the business of the country is, properly, transacted with money, and, while the vital necessity of food renders this conspicuous, it makes no difference, up to the point when extreme and exceptional poverty excludes every other consideration, as to the food, whether too high a price is paid for food or for anything else that virtually is necessary. If, for example, one uses 500 gallons of gasoline a year, and he pays, unnecessarily, 10 cents a gallon more than he did the year before, that $50 difference could be applied on food just as well as a saving of that amount on food, itself; and so on for telephones, telegrams, railroad fares, shoes, taxes and everything else. The farmer or poultry raiser may be getting too much for his labor, and, on the other hand, it is possible that the plumber, electrician and automobile mechanic should not charge more per hour than does the carpenter and the painter. At any rate, a saving, by the ultimate consumer, on any kind of labor will buy him more food, just as well as if the food producer's or food dealer's charges were reduced.

### NEEDFUL STUDY OF FOOD ECONOMICS

All are consumers, not only of food, but of various other services and commodities, and all ought to be, for the greater part of their lives, producers of some service or commodity of a useful nature.

The problem of food economics can not be satisfactorily solved on the theory, often implicity assumed, that it can be separated from the general economics of production and distribution, still less on the assumption that food can be produced and distributed by workers at a less rate than their corresponding degrees of skill and industry are worth in other activities that these workers may actually or potentially be competent to perform. The principle should be recognized that the same degree of skill and industry should have the same pay in any line of work, that an abnormally high pay for any kind of work will tend—quite rapidly in these days—to compensatory increases of wages in similar industries, and that, after or even before, a general increase in wages has been compensated by withdrawal of wealth from amassed capital or abnormally highly paid positions, the result will be a diminished purchasing power of money, so that the increase in wages is fictitious. This means, not merely that the average man must think in higher monetary terms, receiving and expending more than formerly, although breaking even so far as actual standards of living are concerned, but that business and employment will be hampered by a relative reduction of the circulating medium of exchange.

Employment will be reduced, not merely because the prospective employer still is thinking in previously established values and will postpone work until it is absolutely necessary, but, because it is actually difficult to obtain sufficient money or credit, since the amount of money increases but slowly. Unfortunately, while the average laborer is quite inclined to make the pessimistic statement that he is no better off at present than at his former wages, because of the higher cost of living—meaning that the other fellow also is getting higher wages—he can see no other remedy than a further increase for himself, so that we are, in an economical sense, chasing the devil around a stump.

While food can not be separated from more general considerations of economics, it does, as a matter of administrative detail, deserve special attention; indeed, almost every kind of food should be especially and expertly considered with reference to a possible reduction of price or substitution of a cheaper food of equal value.

CAMPAIGN ON FOOD ECONOMICS SUGGESTED

Much of the complaint of the high cost of food could be removed by a popular education in food economics. For practical purposes, a campaign of education should be conducted mainly along three lines; namely:

1. Every consumer of food should know the minimum standard rate (not the occasional bargain price made for a "come along" or owing to a temporary excess of supply over demand) at which a given commodity has been sold in the past or is being sold in a comparable community or one that may be made comparable by scientific attention to methods of transportation and distribution. This will give him an idea of the price goal toward which he may, by constant and united demand, strive. On the other hand, both as a matter of justice and to save him unavailing effort, he should understand whatever real obstacles there exist to the restoring of former prices. Thus, it is obvious, for example, that milk, elaborately protected against infection, bottled instead of vended "loose," produced by regular industry involving large capital and skilled and careful labor, transported to greater average distances, and distributed on an accurate time schedule, can not recede to the price of carelessly handled milk sold as a by-product from a suburban farm. The same argument applies to many other foods.

But even this argument should not be granted without investigation. Many foods can be enormously increased in yield per acre by fertilizing and employing skilled labor, without correspondingly increasing the costs. Potatoes, for example, by intensive methods, may be increased by from 75 to 300 bushels per acre. And, without reference to acreage, the same general principles of efficiency apply to food production as they do to manufacturers.

2. The consumer should also know the current possible low price in his own community. This information has recently been published and the practice should be continued, with explanatory notes, including the general principles of the first consideration.

3. The average consumer is very ignorant of comparative food values, both in the commercial and the physiologic sense. As mentioned in a previous article, much of the most expensive land and labor is devoted to the production of coarse vegetables, that the average person regards as necessary high-food-value articles, but which are of almost no value except as relishes and stimulants to peristalsis. Not only is he paying high prices for virtually nothing, in the nutritive sense, but, his large demand maintains high prices, while, in many instances, there is actual deficiency of nutrition, because of this ignorance.

Failure to allow for gross waste results in a very general buying of the poorer class of meats, at extravagant prices, when the ultimate nutritive value is considered. Barring a few fancy cuts, it is ordinarily true that the kinds of meat sold at the highest prices per pound are the most economical. During the meat strike, a few years ago, enormous quantities of small fish were consumed, at a cost per unit of nutrient considerably higher than that for mammalian meat, against which the strike was conducted, not to mention the qualitative nutritive superiority of the latter. In many instances, the people are actually *mis*-instructed. One can hardly ride in a street car without learning of the high food value and economy of various cereals. No actual misstatement is offered; still, the people do not realize that all that is said of this or that proprietary preparation applies with equal force to standard cereals in bulk and to bread, and that the former cost from two to four times as much as the latter for equivalent food values. An important minor detail requiring careful investigation is the price of crackers. These consist of unleavened bread, they do not involve much waste because of rapid depreciation and should be economic in every sense. It may be that the explanations as to the cost of manufacture are correct; yet they should not be accepted without careful examination by competent, disinterested authority.—*Clinical Medicine.*

